# Low Serum Levels of Zinc and Copper Are Associated With Tuberculosis

**DOI:** 10.7759/cureus.93613

**Published:** 2025-09-30

**Authors:** Priyank Modi, S N Krishnagopal, Rishabh Mittal, Sonu S Ahirwar, Snigdha Singh, Nidhi Chourasia, Ajay Tripathi, Arvind K Shukla, Ashwin Kotnis

**Affiliations:** 1 Medicine and Surgery, All India Institute of Medical Sciences, Bhopal, Bhopal, IND; 2 Biochemistry, All India Institute of Medical Sciences, Bhopal, Bhopal, IND; 3 Biochemistry, All India Institute of Medical Sciences, Rishikesh, Rishikesh, IND; 4 Soil Science, Indian Institute of Soil Science and Research, Bhopal, IND

**Keywords:** copper, micronutrients, serum, tuberculosis, zinc

## Abstract

Background: Tuberculosis (TB) is a curable infectious disease that remains a major health concern in India, causing significant morbidity and mortality globally. Concomitant malnutrition in TB patients, particularly deficiencies in crucial micronutrients such as zinc and copper, is an underexplored area in TB research. These micronutrients play vital roles in immunological defense and are often depleted in TB patients.

Methods: This is a case-control, ethics-approved study that included 50 consenting active TB patients from the Department of Pulmonary Medicine, All India Institute of Medical Sciences (AIIMS) Bhopal, India. Patients were selected based on smear-positive microscopy for acid-fast bacilli or a positive cartridge-based nucleic acid amplification test (CB-NAAT) with a compatible clinical history. Additionally, 50 age-, gender-, and socioeconomically matched healthy controls were enrolled. Venous blood samples were collected and centrifuged, and the serum was quantitatively analyzed for zinc and copper using atomic absorption spectrometry (AAS).

Results: The mean serum zinc and copper levels in TB patients were 59.92 µg/dL and 161.48 µg/dL, respectively, compared to 80.91 µg/dL and 102.74 µg/dL in healthy controls.

Conclusion: Lower zinc levels in TB patients compared with healthy controls highlight the need for zinc supplementation through diet or medication. Further clinical trials are recommended to assess its impact on prognosis and therapeutic outcomes in Central India.

## Introduction

Tuberculosis (TB) is a curable infectious disease causing significant morbidity and preventable deaths worldwide. One-third of the total human population is infected with *Mycobacterium tuberculosis*. This bacterium is responsible for three deaths every minute worldwide. India tops the first five countries ranked in terms of the absolute number of cases [1]. According to the Global TB Report 2021, more than 1.9 million cases with a prevalence rate of 188 cases per 1,00,000 population were reported in 2021 in India, which was 19% higher than the TB cases reported in 2020 [2].

The association between TB and malnutrition was observed long ago. Malnutrition can predispose to TB, and having TB can lead to malnutrition. Malnutrition enfeebles the immune response, which makes people susceptible to infection [1]. Concomitant malnutrition, especially in terms of micronutrient reserves in TB patients, is an under-addressed issue in TB research. Zinc is one of the most important micronutrients depleted from body stores in various inflammatory pathologies, including TB [2,3]. Nutritional status is one of the most important determinants of resistance to infection, and it is well-established that nutritional deficiency is associated with impaired immune functions. There are limited studies that have revealed the correlation between zinc levels and active pulmonary TB in adults, especially in India [4,5]. Zinc is an essential trace element having various functions, such as antioxidant activity, immunological integrity, and cellular immunity [1]. Zinc is important for enzymes of all six classes, as well as transcription and replication factors. It is necessary for the normal function of the immune system [2,6].

Zinc deficiency affects host defense in a variety of ways, and it results in decreased phagocytosis and leads to a reduced number of circulating T cells and reduced tuberculin sensitivity. It also leads to decreased production of interferon-γ, IL-1, and TNF-α. Proposed mechanisms of low serum zinc levels in TB and some other inflammatory diseases include redistribution of zinc from plasma to local tissues, decreased hepatic production of zinc carrier protein named X2 macroglobulin, or increased metallothionein, which increases zinc transport from plasma to the liver. Zinc storage is not adequate, so daily supplementation of zinc is necessary for the normal functioning of the body [1,7].

Apart from the role of zinc in TB, serum copper is also relevant for the prognosis of TB. Estimation of and calculation of the zinc/copper ratio might also be relevant, as copper inhibits the activity of zinc in the body and interferes with the absorption of zinc from the gastrointestinal tract (GIT). Copper, once absorbed into the body, readily accumulates in toxic quantities and inhibits the functions of zinc. The size of the thymus is decreased, and T cells are found to be irregularly functioning or dormant when a copper-zinc imbalance in serum is found. Hence, it has been proposed that zinc should be given in higher quantities as compared to copper, ideally 8:1 in the diet, wherein zinc behaves such as the bigger brother, blocking copper in the food and the body from being absorbed [8]. Hence, the estimation of copper and calculation of the zinc/copper ratio helps find out another parameter to study the interaction of zinc with copper in the human body, as copper inhibits the activity of zinc in the body and interferes with the absorption of zinc from the GIT.

Thus, our study mainly focuses on the estimation of serum zinc levels in the treatment-naive patients of TB. We will also find the interaction of zinc with copper and the outcome of such interactions in tuberculosis patients so that we can devise proper adjuvant supplementation in TB therapy in our further work.

## Materials and methods

Study design and ethical approval

This case-control study was approved by the Institutional Ethics Committee of All India Institute of Medical Sciences, Bhopal (IHEC/STSICMR0161), as well as by the Indian Council of Medical Research, New Delhi, under the short-term studentship (STS) program for medical students. The study enrolled 50 consenting active TB patients, confirmed either by smear-positive microscopy for acid-fast bacilli (AFB) or a positive CB-NAAT (GeneXpert, Cepheid, Sunnyvale, CA) with compatible clinical history, recruited from the Department of Pulmonary Medicine, AIIMS Bhopal, India. In parallel, 50 age- and sex-matched, unrelated, apparently healthy controls were recruited from the hospital premises. This investigation was designed as a preliminary pilot study under the ICMR-STS program, and due to limited time and resources, a modest sample size was used. The primary objective was to identify preliminary trends and generate hypotheses to guide larger, adequately powered studies in the future.

Sample collection and transport

Demographic and clinical data were collected from all participants prior to sample collection. The inclusion criteria for TB cases were adults aged ≥18 years with smear-positive microscopy for AFB or a positive CB-NAAT, along with a compatible clinical history. Additionally, 4-5 mL of venous blood was collected in metal-free vacutainers and transported to the Biochemistry Laboratory under a maintained cold chain for estimation of zinc and copper levels.

Serum zinc and copper estimation

Approximately 5 mL of venous blood was collected from each participant into properly labeled, metal-free tubes containing the appropriate anticoagulant. The samples were centrifuged at 3000 × g for 10 minutes at 4°C to separate the serum. The resulting serum was quantitatively analyzed for zinc (Zn) and copper (Cu) levels using atomic absorption spectrometry (AAS) with a GTA120 graphite tube atomizer (Agilent, Santa Clara, CA) [8]. Additionally, participants’ dietary patterns were assessed using a structured questionnaire administered as part of the study.

Statistical analysis

Demographic and clinical data were recorded in a standardized patient datasheet. Serum Zn and Cu levels in TB patients were compared with those of healthy controls, with control values serving as reference ranges. The Zn/Cu ratio was also calculated to assess the potential interaction between zinc and copper, given that copper can theoretically inhibit zinc activity. Descriptive statistics, including mean and percentage values, were calculated using MS Excel 2010, while p-values for group comparisons were determined by chi-square test using Statistical Product and Service Solutions (SPSS; IBM SPSS Statistics for Windows, Armonk, NY). Statistical significance was set at a p-value < 0.05.

## Results

This case-control study included a total of 100 participants, comprising 50 active TB patients and 50 apparently healthy controls. Among TB cases, 28 (56.0%) were male, and 22 (44.0%) were female, whereas the control group included 30 males (60.0%), and 20 females (40.0%). The age distribution revealed that most TB patients (62.0%) were between 31 and 65 years, 22.0% were 18-30 years, and 16.0% were 66-80 years, while in controls, 50.0% were 18-30 years, 46.0% were 31-65 years, and 4.0% were 66-80 years (Table 1).

**Table 1:** Demographic distribution of TB cases and control participants C-XR: chest X-ray; CB-NAAT: cartridge-based nucleic acid amplification test; AFB: acid-fast bacilli; TB: tuberculosis

Variables	Case N = 50 (%)	Control N = 50 (%)
Gender		
Male	28 (56.0)	30 (60.0)
Female	22 (44.0)	20 (40.0)
Age (in year)		
Range	18-74	18-80
18-30	11 (22.0)	25 (50.0)
31-65	31 (62.0)	23 (46.0)
66-80	8 (16.0)	2 (4.0)
Dietary pattern		
Non-vegetarian	16 (32.0)	27 (54.0)
Vegetarian	34 (88.0)	23 (46.0)
Addictions		
Tobacco	14 (28.0)	12 (24.0)
Alcohol	3 (6.0)	11 (22.0)
Comorbidities		
Hypertension	8 (16.0)	3 (6.0)
Thyroid	7 (14.0)	4 (8.0)
Other	8 (16.0)	4 (8.0)
Family history		
TB	6 (12.0)	4 (8.0)
Diabetes	3 (6.0)	5 (10.0)
Hypertension	12 (24.0)	3 (6.0)
Other	5 (10.0)	5 (10.0)
Detection methods		
C-XR	38 (76.0)	-
CB-NAAT	44 (88.0)	-
AFB smear	12 (24.0)	-
Other	6 (12.0)	-

Dietary assessment indicated a predominance of vegetarian habits among TB patients (68.0%) compared to controls, who were mostly non-vegetarian (54.0%). Tobacco use was reported in 28.0% of TB patients and 24.0% of controls, whereas alcohol consumption was lower in TB patients (6.0%) versus controls (22.0%). Comorbidities, including hypertension (16.0%) and thyroid disorders (14.0%), were more frequent in TB patients compared to controls (6.0% and 8.0%, respectively). Family history of TB and hypertension was also higher in TB cases (12.0% and 24.0%) relative to controls (8.0% and 6.0%) (Table 1).

Regarding TB detection, C-XR was positive in 76.0% of cases, CB-NAAT in 88.0%, AFB smear in 24.0%, and other methods in 12.0% of patients. These findings comprehensively describe the demographic, lifestyle, and clinical profiles of the study population and provide context for subsequent biochemical analyses (Table 1).

**Figure 1:**
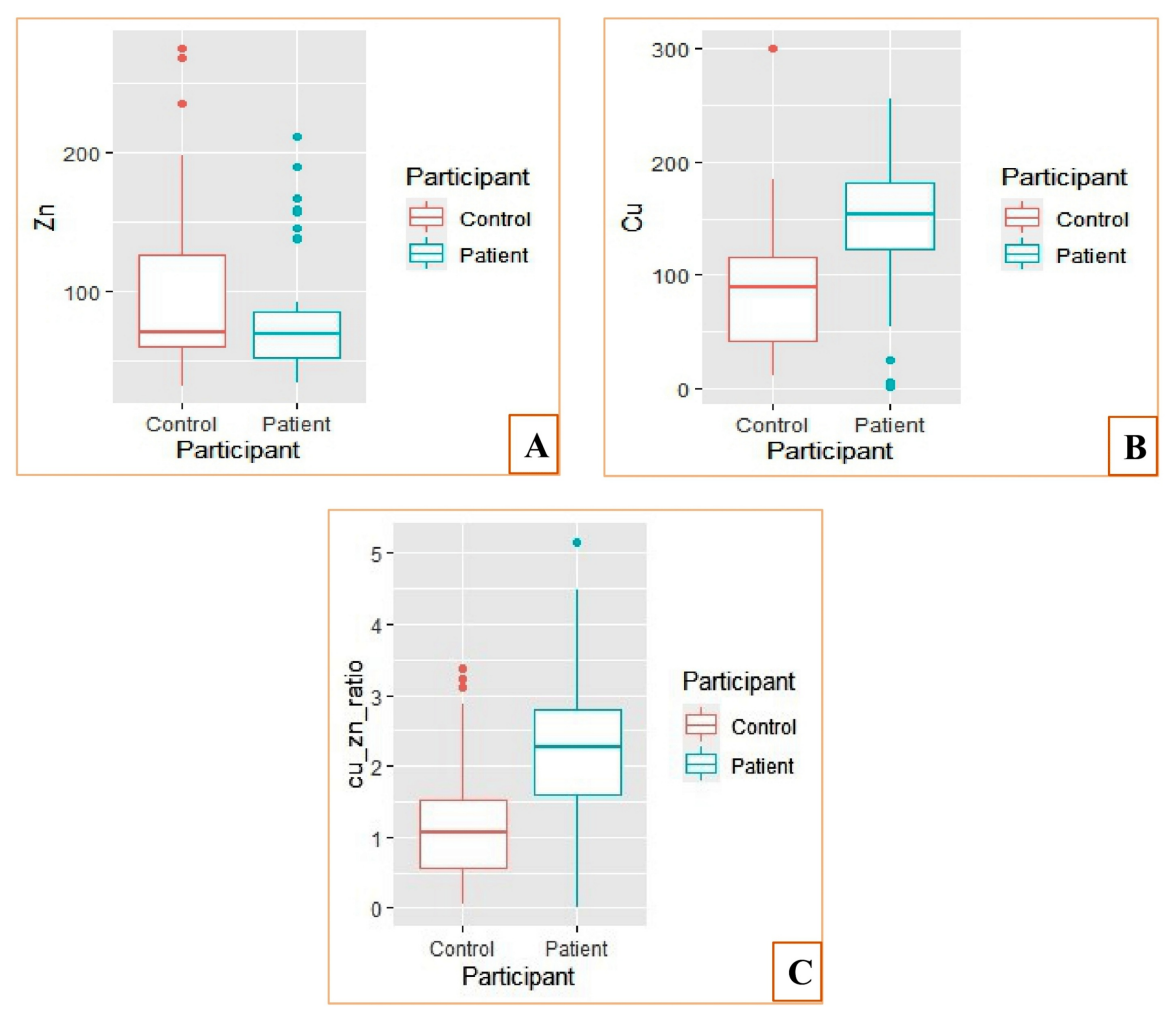
(A & B) Zn and Cu levels in TB cases and controls samples. (C) Zn/Cu ratio

The mean serum concentrations of trace elements in the study population are summarized in Figure 1 and Table 2. In the apparently healthy control group, the mean copper (Cu) level was 90.04 ± 58.35 µg/dL, while the mean zinc (Zn) level was 97.83 ± 54.18 µg/dL. In contrast, TB patients prior to the initiation of anti-tuberculosis treatment exhibited a mean serum zinc concentration of 83.70 ± 59.37 µg/dL and a mean serum copper concentration of 141.90 ± 45.27 µg/dL. We observed that participants with a vegetarian diet tended to have lower serum zinc and copper levels compared to those with a non-vegetarian diet.

**Table 2:** Zinc and copper levels in TB cases and controls

Study Participants		N = 100	Mean Serum Level ± SD	P-value
Zinc	TB Cases	N = 50	83.70 ± 59.37	0.12914
	Controls	N = 50	97.83 ± 54.18	
Copper	TB Cases	N = 50	141.90 ± 45.27	<0.001
	Controls	N = 50	90.04 ± 58.35	

## Discussion

TB is one of the major causes of death throughout the world. In developing countries, especially in India, TB is still one of the major diseases with a higher mortality rate; more than 1.9 million cases of TB were diagnosed in the year 2021. The mortality rate is 37 per 100,000 population. More than 450,000 deaths were reported in the year 2020 in India due to all forms of TB [1,8]. Rising prevalence and mortality are due to poor sanitary conditions, malnutrition, and lack of proper diagnosis [7]. Studies held elsewhere in the world have revealed a controversial relationship between Zn deficiency and TB. Additionally, there is hardly any information available from Central India on this issue [5-7].

The purpose of the present study was to estimate the level of essential trace elements, Zn and Cu, in the serum samples of TB patients and controls of the Central Indian population. These trace elements play an important role in different metabolic processes. However, low Zn level in TB survival was reported in various countries, such as Ethiopia, Iran, Italy, Nigeria, Turkey, Indonesia, Pakistan, and India [4,9-13] (Table 3). The reduced plasma zinc levels observed in TB patients compared with healthy controls may reflect a multifactorial host response to infection. During the acute-phase response, zinc is actively redistributed from the circulation to the liver and other tissues, a process driven by pro-inflammatory cytokines, such as IL-6 and TNF-α. This redistribution is facilitated by decreased levels of zinc-binding carrier proteins (e.g., α2-macroglobulin) and upregulation of zinc transporter proteins, such as metallothioneins, which sequester zinc in hepatocytes and limit its serum availability. This host-mediated sequestration is believed to be part of “nutritional immunity,” restricting zinc to deprive *M. tuberculosis* of an essential micronutrient. Beyond its redistribution, zinc plays a pivotal role in antioxidant defense mechanisms, particularly as a cofactor of the metalloenzyme Cu/Zn superoxide dismutase (SOD), which neutralizes reactive oxygen species generated during immune responses. Zinc is also indispensable for hormonal regulation and both innate and adaptive immune function, supporting thymic hormone activity, T-lymphocyte proliferation and differentiation, and the antimicrobial capacity of macrophages. Consequently, reduced zinc bioavailability during TB may compromise antioxidant defenses and cellular immunity, potentially exacerbating disease progression while reflecting the host’s attempt to limit pathogen access to this critical trace element [14]. Recent studies revealed the importance of Zn in the physiology and normal function of T lymphocytes and macrophages.

**Table 3:** Comparison of available studies of copper and zinc trace elements in tuberculosis patients of different countries

Author (s)	Sample type	Participants		Copper in Mean± SD (µg/dL)		Zinc in Mean± SD (µg/dL)		Country
		TB patients	Controls	TB patient	Control	TB patient	Control	
Nizamani et al. 2019 [11]	Serum	165	171	120 ± 10	82 ± 11.5	91 ± 7.2	112 ± 16	Pakistan
Edem et al. 2015 [9]	Serum	24	20	105 ± 25.0	160 ± 25.3	81 ± 22.1	132 ± 48	Nigeria
Lombardo et al. 2011 [10]	Serum	43	43	173 ± 36.5	166 ± 28.3	59.4 ± 10.9	77 ± 14.8	South Africa
Gulam et al. 2009 [5]	Serum	50	30	-	-	63 ± 5.0	91 ± 5.0	India
Mohan et al. 2006 [4]	Serum	60	60	123.65 ± 9.9	102 ± 20	64.14 ± 3.97	96 ± 18	India

In addition, copper is a fundamental trace element, and it is used as a response to mycobacterium infection. It is a significant constituent of the immune system in opposition to ROS to create a response to TB infection. Although it enhances immunity against macrophage, in the present study, Cu was observed to be greater in TB patients as compared to the control group (Table 3); this finding is also supported by the previous finding [15].

Zn and Cu are the important trace elements for SOD, which is responsible for the synthesis of hydrogen peroxide. Hydrogen peroxide plays a major role in phagocytosis. Consequently, deficiency of Zn/Cu reduces the production of ROS, which is responsible for increased infection vulnerability [11,15].

Limitations

This study has several limitations that should be acknowledged. First, the relatively small sample size (N=50 TB patients and N=50 healthy controls), dictated by the constraints of an ICMR short-term student training project, limits the generalizability of the findings. Second, as a case-control investigation, it provides only a single time-point assessment of serum zinc and copper levels, preventing evaluation of their longitudinal changes during TB progression or treatment. Third, key nutritional parameters, such as body mass index (BMI), detailed dietary intake patterns, and the use of dietary supplements, were not recorded, and inflammatory markers were not comprehensively measured. These omissions restrict our ability to distinguish TB-related metabolic alterations from baseline nutritional deficiencies. Additionally, other potential confounders, such as socioeconomic factors and comorbid conditions, were not extensively analyzed. Future studies with larger, statistically powered cohorts, detailed nutritional and supplement histories, and serial measurements of trace elements and inflammatory markers are required to validate these findings and clarify their clinical relevance.

## Conclusions

Serum copper levels are significantly higher in TB patients compared with controls, consistent with previous studies, and are considered part of a protective mechanism to limit the growth of *M. tuberculosis* in the body. Conversely, the Cu/Zn ratio is significantly lower in TB patients, primarily due to reduced zinc levels. This decline in zinc levels may be attributed to underlying malnutrition in TB patients and/or impaired absorption of bioavailable zinc due to high dietary phytate content.

Our findings highlight the complex interplay between trace element metabolism and TB pathology, suggesting that elevated copper levels may be an adaptive immune response rather than a direct consequence of infection. The absence of a significant association between serum copper and zinc levels and dietary habits indicates that host metabolic regulation may play a more dominant role in determining their concentrations. However, the potential impact of dietary interventions cannot be ruled out. Given the observed imbalance, further research is warranted to explore the therapeutic potential of zinc supplementation and strategies to reduce dietary phytate in TB patients. Future studies should also investigate the long-term effects of trace element modulation on TB disease progression and treatment outcomes to develop more effective nutritional interventions.
